# Horse Sector Participants’ Attitudes towards Anthropomorphism and Animal Welfare and Wellbeing

**DOI:** 10.3390/ani14172482

**Published:** 2024-08-26

**Authors:** Julie M. Fiedler, Margaret L. Ayre, Sarah Rosanowski, Josh D. Slater

**Affiliations:** 1 Melbourne Veterinary School, Faculty of Science, University of Melbourne, 250 Princes Highway, Werribee, Melbourne, VIC 3030, Australia; jmfiedler@student.unimelb.edu.au (J.M.F.); jdslater@unimelb.edu.au (J.D.S.); 2 School of Agriculture, Food and Ecosystem Sciences, University of Melbourne, Royal Parade, Parkville, Melbourne, VIC 3010, Australia; mayre@unimelb.edu.au

**Keywords:** anthropomorphism, horse, equine, equestrian, horse racing, animal welfare, animal wellbeing

## Abstract

**Simple Summary:**

Adopting contemporary animal welfare and wellbeing assessment methods requires making inferences about psychological experiences, a process at risk of anthropomorphic bias. Respondents to an online survey were asked for their opinions on how anthropomorphism could relate to horse wellbeing. The results suggest that experienced horse sector participants were aware of the effects of anthropomorphism, including its potential beneficial and detrimental impacts on horse welfare and wellbeing. This included the beneficial effect of motivating individuals to care for horses and the detrimental effect of misinterpreting horse behaviors. The authors propose that anthropomorphism has a place in horse wellbeing when used critically and with caution. Horse-related organizations should actively manage anthropomorphism in policies and practices to avoid compromising animal welfare and wellbeing practices.

**Abstract:**

Anthropomorphizing can misinform the making of inferences about animal mental experiences. This is a consideration when implementing the Five Domains Model for animal welfare assessment. An online survey run in 2021 captured horse sector participants’ perspectives about anthropomorphism and wellbeing in relation to horses. Most respondents, 82.9% (*n* = 431/520), believed that anthropomorphism could influence horse welfare and wellbeing. These respondents were then asked how, in their opinion, anthropomorphism might relate to horse welfare. A thematic analysis identified two themes: (1) ‘Anthropomorphism can influence how people relate to horses’ and (2) ‘Anthropomorphism can have consequences for horse welfare and wellbeing’. The results suggested that experienced respondents were aware of the complexities surrounding anthropomorphic attitudes and that anthropomorphism can have beneficial and detrimental consequences on horse welfare and wellbeing. Benefits include a sense of relatedness to a horse, while detriments include the potential to misinterpret horse behaviors. The authors propose that anthropomorphism has a place in horse welfare and wellbeing when used critically and with caution. This study recommends that there is a need to actively manage anthropomorphism when organizations update policies and practices and implement the Five Domains Model. More research is required to understand the effects of anthropomorphism on horse-related practices.

## 1. Introduction

Anthropomorphism is when people attribute or project a human-like mind or human-like physical characteristics onto nature, an animal, or an object. [1,2]. The phenomenon is inherently a human trait, a unique experience for each person, and a lifelong tendency [1,3,4]. Anthropomorphism is used in marketing campaigns to attract consumers to engage with various products and causes, such as personal technology, pet products, and wild animal conservation initiatives [5,6,7]. The range of animal species anthropomorphized is diverse, encompassing wildlife and domestic animals such as horses, dogs, and birds [6,8,9,10].

Anthropomorphism is important because contemporary approaches to animal care and the assessment of welfare and wellbeing require making inferences about an animal’s current mental status [11,12]. It has been recognized that there is a risk that an anthropomorphizing individual may misinterpret animal behaviors, which subsequently might misinform inferences and assessments of an animal’s welfare and state of wellbeing [11,13]. Another factor relates to the interchangeable use of the terms animal ‘welfare’ and animal ‘wellbeing.’ Smith et al. [14] discovered that horse owners do not differentiate between these terms and construct meaning in the context of their own experiences and knowledge of horses. The current study adopts both terms, with the term welfare recognizing that welfare is a state within an animal and that wellbeing, defined by Simons and Baldwin [15], is “meeting full potential in the world and possessing a positive mental state”.

Scientific disciplines which have a technical focus on objective measurements have traditionally avoided applying anthropomorphism because its use is associated with subjective methods and with attributing human cognitive properties to animals [16]. Not all authors agree with denying anthropomorphism, arguing that scientists perceive their work through human eyes and that a natural bias towards anthropomorphism exists; therefore, achieving complete detachment is unlikely [16,17,18]. Further, avoiding anthropomorphic language when reporting scientific findings may not be enough to claim objectivity unless the researcher’s anthropomorphic tendencies have been actively managed [19,20]. Acknowledging anthropomorphism can open up opportunities for scientific discovery and new ways to understand human–animal interactions [17,18,21]. When applied with caution, which involves critical evaluation of anthropomorphic interpretations of animal behaviors with evidence-based information from multiple sources, anthropomorphism may inform the development of new theories, a cautious inference, or a testable hypothesis [11,16,17]. Critical approaches can assist in managing risks associated with anthropomorphic bias when inferring animal mental experiences [11,17]. By problematizing how humans perceive animals and their welfare and wellbeing state, biases like anthropomorphism and related concepts, such as anthropocentrism, can be managed although not entirely eliminated [16,17,22].

There is no single approach to describing or using anthropomorphism [2,23]. One common outcome of its use is as an explanatory effect, such as explaining that a dog’s behavior was motivated by spite [23]. Another is when anthropomorphizing results in humans personifying animals, whereby humans think about animals as they think about themselves, a phenomenon reported to occur among horse owners [8,24]. Critical anthropomorphism is the conscious, applied use of anthropomorphism as a preliminary step when developing a testable hypothesis or making a cautious inference about an animal’s mental experience [16,25]. For example, when making an inference, an individual observes animal behaviors and actively manages anthropomorphic bias by using reflexive practices when making a cautious inference [11,18]. The inference is critically evaluated by referring to science and other evidence, such as a carer’s personal knowledge about the animal, and then reviewed using reflexive practices [11,16,18]. This approach should minimize possible interference with the established scientific processes of data collection, analysis, interpretation, or other methods for assessing and reporting on animal welfare and wellbeing [16,22].

Critical anthropomorphism has also been an approach identified in relation to some animal-related government policies [26]. Caulfield [26] argues that elements of critical anthropomorphism are evident in The Brambell Report [27], a technical document that led to the development of the Five Freedoms paradigm [28,29], which guided approaches to animal management by setting freedom from (1) hunger and thirst, (2) physical discomfort, (3) injury and disease, and (4) fear and distress and by (5) providing animals with the freedom to express behaviors [28]. The Brambell Report states that “The evaluation of the feelings of an animal similarly must rest on an analogy of our own.” This is before stating that there is a need for a comparison of personal views with scientific knowledge and the making of a balanced judgment [27].

The prevalence of anthropomorphism among animal carers [30,31,32] contrasts with the avoidance of anthropomorphism in animal science [16]. At an individual level, anthropomorphizing may give some people a sense of control over their surroundings and when interacting with an animal, a sense of predictability regarding the animal’s behavior [1,3]. Its use may also allow some individuals to gain a sense of personal identity by positioning their favorite animal within family and social networks [1,6,33]. When investigating the human–horse bond, Merkies et al. [31] reported that some owners anthropomorphized the relationship with their horses; for example, one owner described a particular horse as motivated to play tricks or to laugh at them. Anthropomorphic effects may not always be evident because of cultural, biological, and situational factors, such as the tendency to gender stereotype animals [1,22,34]. An example of anthropomorphism in horse racing’s cultural context is the social positioning of racehorses as heroes [35]. Dashper et al. [36] found an anthropomorphized preference for male horses over female horses and the labeling of female horse behaviors using terms such as moody. Other anthropomorphic attitudes may be easier to identify, for example, when dogs are dressed in fashionable clothing by people [6].

Anthropomorphism can influence decision making by animal carers about management and training practices with either detrimental or beneficial outcomes for animal wellbeing. Its use has been implicated in the provision of inappropriate living conditions for pet birds [9], dog training that resulted in disruptive behaviors [37,38], and the over-blanketing of horses, with the risk of thermal discomfort [39]. Anthropomorphizing animal carers might also make management or training decisions based on personal values rather than the animal’s requirements, with the consequence that they inhibit the animal’s expression of agency [12,40]. For example, a personal preference for cleanliness projected onto horses can influence reasoning for confining horses in stables, a housing option that restricts horse’s behavioral choices, such as rolling [41]. These consequences can extend to holding animals responsible for their behavioral choices and justifying punishment, such as blaming a horse for expressing defiant behavior and responding with overuse of the reins and spurs [42]. When used with caution, anthropomorphism could benefit animal wellbeing when incorporated into communication strategies to engage people in the topic of animal wellbeing and welfare [22,30,43]. For example, a client may use anthropomorphic language during a veterinary clinical examination to describe their pet’s health and behaviors [44,45]. When communicating treatment options to the client, the veterinarian may need to accommodate the client’s anthropomorphic tendencies and use these effects to facilitate the client’s receptiveness and understanding of the proposed treatment plan [9,44,45,46]. As the consequences of anthropomorphism on animal welfare and wellbeing are not always known, its use in all animal-related situations requires careful consideration [22,47]. The aim of this study was to gain insight into the perceptions of experienced horse sector participants about anthropomorphism and its relationship to horse welfare and wellbeing. The objectives were to determine whether participants believed that anthropomorphism could influence horse welfare and wellbeing and, if so, to gather practical examples of those influences. Respondents perceived that anthropomorphism could have both beneficial and harmful effects on horse wellbeing. Respondents also recognized the importance of understanding and managing the impacts of anthropomorphism in the sector. This study therefore addresses a gap in knowledge related to understanding and managing the impacts of anthropomorphism in horse management, training, and performance practices.

## 2. Materials and Methods

### 2.1. Study Description and Sampling Frame

**Figure 1:**
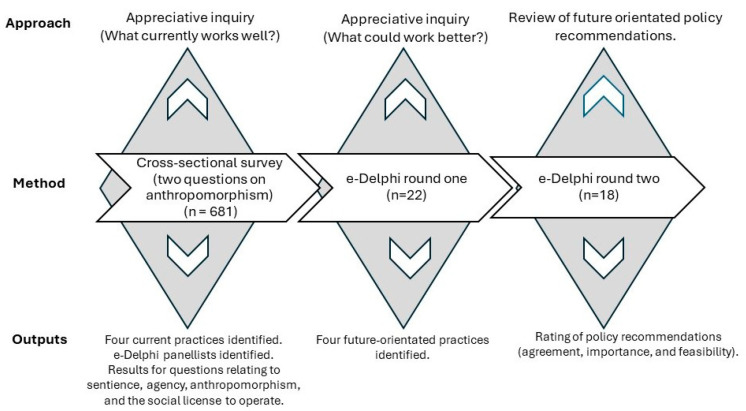
The Futurehorse project: activities and outcomes. The Futurehorse project is a mixed-method study investigating horse sector participants’ attitudes regarding practices for horse wellbeing. The study utilized two data collection methods: a cross-sectional survey and a two-round e-Delphi. Each diamond represents a data collection point. The survey contained two questions on sentience. This study reports the results.

This study reports the results from two questions about anthropomorphism contained within a larger cross-sectional survey conducted as part of the Futurehorse project (Figure 1). The aim of the Futurehorse project was to investigate the perceptions of horse sector participants about future-orientated practices for horse welfare and wellbeing. The survey was open for 57 days in July and August 2021. Individuals self-assessed as meeting the following criteria: citizens of Australia or the United Kingdom (UK), over 18 years of age, and having three or more years of experience in horse racing, riding, sports, or tourism and interested in donkey, horse, or mule welfare and wellbeing.

### 2.2. Conceptual Framework

The theoretical perspectives employed combined Interpretivism with those of animal affective states, biological functioning, and natural living, the latter three of which underpin the approach to the Five Domains Model [48,49,50]. The Interpretivism perspective acknowledges that an individual’s viewpoints about horses, anthropomorphism, welfare, and wellbeing are context-dependent and influenced by their worldviews, encompassing personal beliefs and their experiences with horses [49,50,51]. The animal-centric Five Domains Model consists of a framework outlining physical and psychological indicators to guide the systematic assessment of animal welfare by focusing on four domains: the animal’s physical environment, health, nutrition, and behavioral interactions contributing to the fifth domain, animal mental experiences [13]. These perspectives informed the research processes, including the study design, analysis, interpretation, discussion, and articulation of how the study findings are transferable for the horse sector.

### 2.3. Data Collection

The Futurehorse survey, hosted on the Qualtrics [52] platform, consisted of 35 questions presented in two sections. The first section contained demographic questions followed by pairs of closed and open questions about sentience, agency, and anthropomorphism and a question about the social license to operate. The second section contained 18 questions asking which horse management practices are currently performed well, 12 of which were open-ended, and 2 questions asking for general feedback on the survey experience. The data presented in this paper are from the pair of questions about anthropomorphism in the first section of the survey. The results of the questions about sentience and agency and the results from the second section of the survey will be reported in subsequent publications.

The first question about anthropomorphism was close-ended, which asked respondents whether anthropomorphism could influence horse welfare outcomes. The response options were yes, no, or do not know. If a yes response was received, the survey progressed to the second open-ended question, asking respondents to consider how anthropomorphism could relate to horse welfare. If a no or do not know response was received, the survey’s skip logic directed respondents to the next section of the questionnaire, skipping the open-ended question. The survey included a description of anthropomorphism as “the attribution of human behaviors or characteristics to animals” to improve the consistency of responses [53].

### 2.4. Analysis

**Figure 2:**
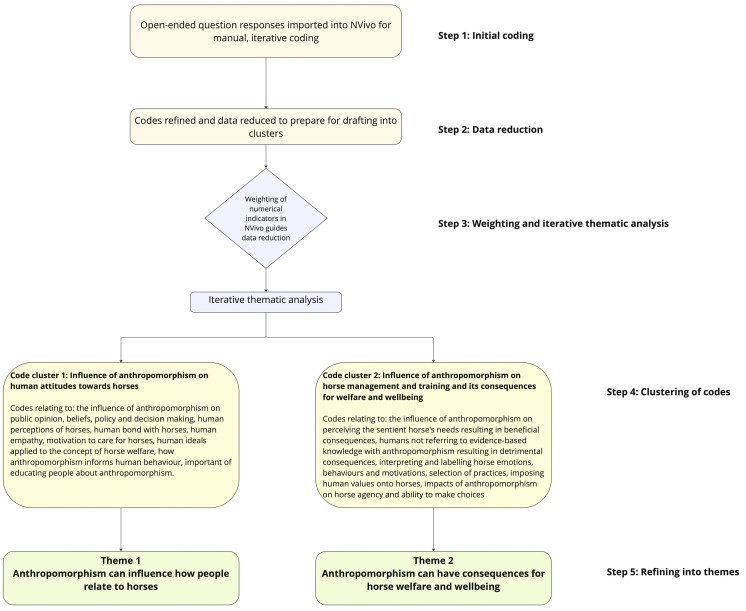
Coding methodology. In the first step, raw data were imported into NVivo [54] for manual iterative coding, employing inductive, deductive, and abductive reasoning processes [55]. Step 2 involved refining codes and reducing data for drafting into clusters. In Step 3, data were weighted using numerical indicators in NVivo in preparation for the iterative thematic analysis. Steps 4 and 5 involved forming initial clusters and then refining these into the final themes.

The Futurehorse project and this study utilized the following research processes. Survey responses were extracted from Qualtrics, and each response was allocated a case number. The data file was cleaned to remove ineligible responses, such as those that did not answer at least one question following the demographic question set. Eligible responses were categorized according to country, age, gender, equid species, amateur or professional status, job role, and years of experience within the industry. Quantitative data were analyzed with Microsoft Excel and reported as descriptive statistics (count and percentage). As the number of responses for each question differed, the denominator varied. Before the qualitative analysis commenced, the lead author revisited the research questions and read the responses to gain familiarity with the data set [54]. Referring to Braun and Clarke (2022) [55], a five-step analysis process was undertaken to determine the final themes (Figure 2).

The co-authors utilized reflexive practices, described as continuous, critical self-reflection about the researcher’s influence on the research process and, more widely, animal welfare and the societal context to which the study relates [56]. The reflexive techniques incorporated group discussions, whiteboards to refine themes, and critical feedback. Quotes from the raw data were included to exemplify themes when reporting the qualitative results. Square brackets inserted into quotes indicate where text was inserted to improve readability.

## 3. Results

### 3.1. Demographic Characteristics

Of the 681 people who completed the Futurehorse survey, 520 responses were received for the close-ended question relating to anthropomorphism and horse welfare (*n* = 520/681; 76.35%). Of these, 431 (82.9%) agreed that anthropomorphism could influence horse welfare and wellbeing and were, therefore, eligible to participate in the current study. The majority of respondents were Australian (*n* = 393/431; 91.2%) and female (*n* = 367/431; 85.2%). Participants were aged between 18 and 39 (*n* = 108; 25.0%), between 40 and 49 (*n* = 78; 18.1%), between 50 and 59 (*n* = 124; 28.8%), or 60 or over (*n* = 121; 28.1%). Nearly half (*n* = 188/397; 47.4%) considered themselves professional and almost all (*n* = 414/426; 97.2%) engaged in horse-related activities. The majority of participants had more than 20 years of experience in the horse sector (*n* = 381/431; 88.4%). In total, 46.8% (*n* = 173/370) of participants were involved in equestrian sports, 12.2% (*n* = 45/370) in horse racing, and 10.0% (*n* = 37/370) in other competitive activities; 7.3% (*n* = 27/370) were involved in multiple activities and 6.5% (*n* = 24/370) in recreational activities. The remaining participants were in ‘other’ or unclassified equestrian activities. In total, 46.7% (*n* = 170/364) of participants considered their role as an activity manager, 15.9% (*n* = 58) were in horse management or training, 14.6% (*n* = 53) were in other roles, such as a general volunteer or media, and 5.8% (*n* = 23) considered themselves as participants only. There was no difference in the demographic characteristics of those who agreed with the close-ended question regarding anthropomorphism and those who disagreed.

### 3.2. Thematic Analysis

The results showed that experienced horse sector participants recognized that anthropomorphism could influence an individual’s attitude towards horses and horse management practices with varied outcomes for animal wellbeing. An analysis of the qualitative responses to the open-ended question identified two themes. The first theme, ‘Anthropomorphism can influence how people relate to horses‘, is focused on the effects of anthropomorphism on the public opinion of horses and how individuals connect with horses. Theme Two, ‘Anthropomorphism can have consequences for horse welfare and wellbeing’, is focused on anthropomorphism as having either beneficial effects on horse welfare and wellbeing, such as motivating an individual to provide care, or having adverse effects that could lead to harm, such as punishing horses.

### 3.3. Theme One: Anthropomorphism Can Influence How People Relate to Horses

The first theme focused on the presence of anthropomorphism in society and its potential to affect public opinion and decision making in organizations. There was a perception that anthropomorphism could affect how an individual relates to a horse and motivates them to provide care. Respondents identified the potential to use anthropomorphism to influence changes in human behavior towards horses.

Respondents believed anthropomorphism was prevalent in society and “social media” (C 874). There was an awareness that “more people care about and relate to animals through anthropomorphism than without it” (C 400) and that the phenomenon may generate emotional responses such as “guilt” (C 697) or “empathy” (C 92) and could “affect public opinion” (C 721) about the involvement of horses in sports and other activities. Respondents expressed concerns that anthropomorphizing members of the public might “believe that horses do and think like humans” (C 842) and recognized that some individuals with anthropomorphic perceptions are “unfortunately influential enough to apply pressure to authorities” (C 1140), including within animal law and policy. These respondents explained their concerns:

“The public has a loud voice and can influence regulations affecting equestrian sports, and there is a tendency to anthropomorphise any animal these days; people don’t like to see animals used in activities that can be seen as entertainment for humans.”

(C 360: equestrian sports, amateur)

“It [anthropomorphism] can undermine legal investigation prosecution and make welfare decisions unclear.”

(C 960: equestrian sports, professional)

Anthropomorphism was also perceived to affect the attitudes of individuals attending horse-related gatherings such as committees and working groups, posing a risk that decisions about welfare and wellbeing could be “made on [a] poor scientific bases” (C 76). For example, one respondent said the following:

“The people involved with making decisions or influencing public policy can be misled in their endeavours to optimise horse welfare because of anthropomorphic judgments.”

(C 867: horse racing, professional)

Respondents were aware that situations might arise where individuals who possessed knowledge about horses could be “overruled by those expressing anthropomorphism” (C 580) or, conversely, where anthropomorphism “is thrown around in a pejorative way to denigrate people who recognize sentience in animals” (C 94). Respondents acknowledged that it was not always possible to know what impact anthropomorphism might have on horses’ wellbeing but considered it essential to be cognizant of its effects:

“Anthropomorphism is often used as a tool of persuasion when animal welfare is discussed at a community level.”

(C 108: amateur)

Respondents recognized that anthropomorphism could shape an individual’s opinions about their relationship with their horse. The attribution of “human behaviour characteristics” (C 1118) to a horse could, for some people, make the horse more “relatable” (C 684). For example, one respondent stated the following:

“Some humans relate to their horses and pets as almost being human.”

(C 417: other horse sports, amateur)

Respondents perceived that “through their own behaviours” (C 972), individuals could perceive a “closer bond and connection” (C 796) with a particular horse and used terms of endearment such as “fur children” (C 711). For example, one respondent stated the following:

“I believe that anthropomorphism at its most basic is actually a protective shield to minimise cruelty, abuse, and neglect. As we assign human characteristics to an animal, we are more likely to treat it with the same degree of empathy and concern that we would a human baby or child.”

(C 147: equestrian sports, amateur)

Respondents recognized that such attitudes might also motivate an individual to provide “superior” (C 840) care for a favored horse and provide every positive experience “that they can afford” (C 1152). They also recognized that such an anthropomorphized perception of closeness to a favorite horse might “keep people honest in [the] treatment of animals” (C 1079), acting to motivate them to keep a horse safe from harm:

“In some ways, it [anthropomorphism] increases animal welfare as people are more likely to spend time and money on animals they feel [a] greater human attachment to.”

(C 93: equestrian sports, amateur)

Respondents also believed that anthropomorphism could misinform an individual’s assessment of a horse’s wellbeing through the “tendency to think of welfare from their own perspectives rather than an animal’s” (C 993). In such situations, horse behaviors indicative of pain or stress could go unrecognized by anthropomorphizing individuals, as this respondent explains:

“Believing that horses have the same brain, thought [and] reaction as humans is dangerous as key signs and indicators that should be recognised for horse pain and distress will be ignored.”

(C 1000: other horse sports, amateur)

Respondents also perceived that these misinformed assessments could extend to judgments about a horse’s quality of life because these were based on “human ideals of comfort” (C 854). Two quotes in particular captured this sentiment. The first related to a decision many horse carers will face, which is when to euthanize their horse due to physical or psychological pain or old age; however, anthropomorphic effects can negatively affect decision making—although these effects were not explained. The second quote provided some insight, implying that what constitutes a good or quality life from a human perspective will not be the same for a horse:

“Anthropomorphism affects our end-of-life choices for older horses or horses in pain and distress.”

(C 1125: equestrian sports, professional)

“It [anthropomorphism] can be a negative as people confuse longevity with quality of life.”

(C 1082: amateur)

Respondents were aware of how anthropomorphism could impact the interpretation of horse wellbeing. For such reasons, they considered the potential of anthropomorphism to influence human behavior change and for educational purposes because it might “get people thinking” (C815) about how they treat horses and how they “decide [about] a horse’s welfare” (C945). For example, one respondent stated the following:

“If this information [anthropomorphism] was more understood by coaches and riders [as] part of the coaching curriculum, horses would suffer far less.”

(C 1053: equestrian sports, professional)

“We need to take into account how people perceive or view horses and other companion animals to alter or influence the human’s behaviour in [that] correct welfare decisions can be made through the lens of anthropomorphism.”

(C 135: equestrian sports, amateur)

### 3.4. Theme Two: Anthropomorphism Can Have Consequences for Horse Welfare and Wellbeing

The second theme focuses on how anthropomorphism could have beneficial and detrimental effects on horse management, training, performance practices, and welfare and wellbeing. Perceived benefits included situations where anthropomorphizing raised an individual’s awareness of horse emotions and needs and could influence the provision of better horse management practices. Conversely, there was a perception that anthropomorphism could have detrimental consequences if an individual inappropriately held a horse accountable for behavior choices and, in some situations, used the judgment to justify the punishment of the horse.

Respondents acknowledged that anthropomorphism could have benefits if an individual viewed horses as “sentient” (C 836), and it raised awareness that animals could “communicate their feelings” (C35) through expressing behaviors:

“The human would need to be self-aware enough to own their projections horses are not humans; they have their own needs, and they must be understood through the lens of being a horse, not how a human might perceive a situation they absolutely do have their own internal life and emotions.”

(C 537: therapy, professional)

They recognized that in some situations, anthropomorphizing might help individuals become more attuned to horses and “conscious of their needs” (C 1118) and might motivate people “to make good decisions about [horse] welfare” (C 1005) and aim to “give them [horses] a better life” (C 1134). However, respondents were also cautious, believing it was important to “not to be slaves to anthropomorphism” (C 377) and, importantly, to “master the boundaries” (C 628) between anthropomorphic perspectives and science. This respondent explained the following:

“It [Anthropomorphism] can lead to poor outcomes [for horses] when the anthropomorphism does not enable the decision maker to better understand the horse’s needs. It can lead to better outcomes when it enables the decision maker to make objectively better welfare decisions.”

(C 108: amateur)

Respondents believed anthropomorphism could pose a risk because it made “humans less critical and less clinical” (C 516) when making decisions about horses. Respondents also believed if an individual thought “anthropomorphically rather than species centrically” (C 155) and did not refer to “science and ethology” (C 1053), there was a risk that decisions relating to horse management practices would not be “evidence-based” (C 1146), with adverse outcomes for horse wellbeing. They felt that an anthropomorphizing individual might fail to consider the “equine perspective” (C 21) and “disregard the natural biological processes of the horse’s body” (C 195), resulting in misinterpretation of the horse’s requirements. Instead of anthropomorphizing, respondents believed it was essential to “honour the horse’s true nature” (C 594), to consider equine “feelings” (C 366), and to let “the horse be a horse” (C 821) because such perspectives provided better outcomes for horse welfare and wellbeing. The following are the words of one participant:

“[Anthropomorphism] can be a negative. Putting human thought processes on the horse could be overriding the horse’s actual needs.”

(C 1042: horse racing, professional)

Another risk respondents recognized related to the interpretation and labeling of horse behaviors and motivations is when “stories are attached to [horse] behaviours” (C 220). Respondents provided examples of labels for behaviors and motivations, which included the terms “regret” (661), “revenge” (C 1115), “malice” (C 668), “sly” (C 1133), “naughty” (C 347), “stubborn” (C 1008), “retaliatory” (C 961), “lazy” (C 627), or “happy” (35). One respondent exemplified this when they commented that people could use anthropomorphization to generalize about horses as embodying human qualities:

“Myths and misconceptions. It is chestnut and a mare; therefore, it must be hot and sensitive.”

(C 519: equestrian sports. professional)

Respondents acknowledged that some individuals may attribute “human moral agency” (C 165) to horses and hold horses accountable for their behavior choices. For example, one respondent noted that people can rationalize horse behaviors based on human traits or tendencies:

“Too many riders seem to think horse deliberately plans to take [the] wrong lead on purpose easier to blame horse than themselves.”

(C 1054: recreation, amateur)

They also recognized that some individuals might think horses “understand and care for human constructs” (C 968) and might conceptualize situations where horses “act, feel and behave” (C 101) in the same way as people. Respondents perceived individuals may subsequently interpret a horse’s motivations and behaviors as “deliberately intentioned” (C 693) or “deliberately obstructive” (C 64) and assign blame because they believe the horse “knows what it is supposed to do” (C 115). This respondent explained with the following:

“Many humans do believe that a horse has deliberately acted to exact bad behaviour so as to harm a human.”

(C 872: equestrian sports, amateur)

Respondents also believed anthropomorphism extended to individuals justifying “decisions on reward or punishment” (C 304) for the horse:

“People place blame on horses and may give the horse credit for pre-planning bad behaviour; therefore, they choose to punish the horse.”

(C 199: other horse sports, amateur)

They acknowledged an individual could judge a horse’s behaviors and attribute blame, for example, declaring the horse was “doing it on purpose to annoy me” (C 564), as one respondent described:

“I often hear riders assigning human reasoning ability to horses and blaming and punishing them for certain behaviours way after the event as if the horse would know what it was supposed to do and relate the punishment to the crime.”

(C 1053: equestrian sports, professional)

Conversely, respondents recognized that in some situations, an individual might perceive that a horse should have behaved in a certain way but did not express an expected behavior, such as showing “gratitude” (C 1008). Respondents were aware that anthropomorphizing “could influence the actions we take or the things we expect from them [horses]” (C 944) with consequences that are “more dangerous than beneficial” (C 391), including punishing horses:

“Should a horse not show the appropriate human anthropomorphic response, i.e., guilt, anger, fear, acceptance, the horse may be inadvertently punished for this.”

(C 800: equestrian sports, amateur)

Another negative consequence of anthropomorphism for horse wellbeing, as perceived by respondents, related to denying horses the opportunity to make meaningful choices:

“People make decisions for horses based on how they feel or how they want the horse to feel, but would that be the horse’s choice [?].”

(C 105: other horse activities, professional)

Respondents perceived there was a risk that the horse would suffer because horses have “completely different reasons for the choices, actions they have made” (C 309).

Opportunities for horses to make choices can be negatively impacted by anthropomorphizing, as these respondents explained:

“I think it [Anthropomorphism] may cause people to give horses more credit for agency than they might actually have, [but] it risks the assumption that the horse is operating on a similar mental level to humans, and that’s just not the case.”

(C 973: equestrian sports, professional)

“By assuming that horses value the same things as us, we are at risk of limiting their agency.”

(C 555: horse racing, professional)

Respondents also acknowledged that anthropomorphism is inherent in some traditional horse care and management practices, although individuals undertaking these practices may not be aware of its occurrence. They believed some individuals “cannot identify anthropomorphism as a framework for their own practices” (C 820), and situations could arise where bias led to “adverse husbandry horse practices” (C 403). Some examples given included “rugging horses because we are cold, three meals a day with nothing in between because that’s what we do” (C 619) or providing a horse with a “duvet day when the horse would rather be allowed out to be a horse” (C 391). One respondent stated the following:

“People put their value on things such as the horse being happy, rugged and stabled when their natural behaviour is to browse and graze twenty-four hours a day.”

(C 159: other horse sports, amateur)

Respondents also perceived that anthropomorphic bias could influence how some individuals explain the involvement of horses in sports, such as attributing ambition to a horse and justifying practices occurring in these contexts:

“All horse sports, and most horse management, greatly rely on human anthropomorphism to create an attitude that horses enjoy their work, way of life, etc. The facilitators of these horse events may often believe themselves that horses love living alone in a stable, travelling around in lorries [and] planes, with limited turnout, learning unnatural gaits, head positions, etc.”

(C 284: equestrian sports, professional)

These care and management practices included “riding [and] competing in adverse weather” (C 1136) or “over-rugging or extreme presentations in showing” (C 442). If individuals accepted such anthropomorphized attitudes and practices as normal, respondents believed it could result in adverse outcomes for horse wellbeing:

“Ascribing horses with human emotions or wants and needs can mean that horse owners overlook species-specific needs, for example, feeling that horses desire to be successful at competitions and value training and exercise above a more natural way of living.”

(C 1125: equestrian sports, professional)

## 4. Discussion

This study investigated the attitudes of experienced horse sector participants about anthropomorphism and horse welfare and wellbeing. The results showed that anthropomorphism was perceived as prevalent in the horse sector and could affect decision making, policy, and management practices. An analysis of qualitative data on the concept and use of anthropomorphism identified two themes: the first considered how anthropomorphism could influence how people relate to horses and how they form opinions, and the second considered the consequences of anthropomorphizing for horse welfare and wellbeing. Respondents recognized that anthropomorphism manifested differently within individuals and in horse management practices, with outcomes ranging from beneficial to harmful for horses. Respondents believed managing risks associated with inferring horse mental experiences was possible by remaining cognizant of anthropomorphic bias. The authors propose an alignment framework to support horse-related organizations in promoting the recognition, management, and monitoring of the effects of anthropomorphism among stakeholders.

In Theme One, the results showed an awareness amongst respondents that anthropomorphism could influence public opinion and a concern that these attitudes could pose a risk to the ongoing public support for horse-related activities. This finding resonates with studies reporting the use of anthropomorphism to gain public support for animal welfare and wellbeing initiatives and for animal rights causes [57,58,59]. It also resonates with studies implicating anthropomorphism in the public shift away from attitudes about domination and control of nature and animals towards a sense of mutualism encompassing the compassionate consideration of nature and animals [10,11,12,13,14,15,16,17,18,19,20,21,22,23,24,25,26,27,28,29,30,31,32,33,34,35,36,37,38,39,40,41,42,43,44,45,46,47,48,49,50,51,52,53,54,55,56,57,58,59,60]. Describes the mutualistic human–mule relationship as featuring listening, reciprocity, and continuous dialogue. In such a relationship, the human moves away from an attitude of control over an animal to focus on co-creating experiences [61]. The results also found that anthropomorphic attitudes were perceived to impact decision making about horse management in policy settings. Anthropomorphism has been raised as an item of consideration and concern in studies about wildlife because it can influence public values about animals and change expectations about wildlife management regimes, such as seeking alternatives to killing nuisance wildlife [10]. Manfredo et al.’s (2020) results provide insight into other animal sectors where public attitudes influence policy, for example, the contested policies of killing wild horses in Australia [62] and the erosion of public tolerance for dogs killed when racing [63,64]. Studies about the horse racing sector report anthropomorphic attitudes among anti-racing advocates, positioning horses as victims, and among pro-racing advocates who position horses as animals that love to race [65,66]. The anthropomorphic effects on policy-making and public attitudes about horse-related activities, which could impact the social license to operate, remain understudied.

Theme One results also showed that respondents were aware that individuals socially positioned horses as an extension of themselves or as a family member, exemplified by using terms such as child and baby. The results also provide insights into how some individuals perceived relationships with horses, such as increased empathy and motivation to care for them. The results resonate with studies reporting similar anthropomorphic attitudes toward dogs [6] and birds [9] and with studies reporting how anthropomorphic attitudes are inherent in the way horse owners talk about and situate their relationship with their horses and orientate decision making about management practices [8,31,67,68]. The results of this study suggest there is a risk that anthropomorphizing can misinform a carer’s perception of the horse’s wellbeing state, including when to blanket a horse or when to euthanize, which is a finding commensurate with other studies [9,39,67,69]. These examples of anthropomorphic bias, which might result in adverse outcomes for horse welfare and wellbeing, can be addressed by applying the Five Domains Model and referring to scientific evidence to guide decision making [13]. If necessary, an animal welfare expert can assist the owner in holistically applying the Model by evaluating relevant indicators in the domains of health, nutrition, physical environment, and behavioral interactions and making an inference about the horse’s mental status [13,39,70].

This study’s results suggest that in some circumstances, behaviors indicative of pain or other negative mental experiences might be anthropomorphized and, subsequently, normalized or ignored. The potential for anthropomorphic bias to misinform behavioral interpretation and management practices has been considered regarding negligence [9] and delayed euthanasia [69,71] among pet-keepers. Behaviors are an indicator when assessing a horse’s quality of life, with behavioral indicators of pain and repetitive behaviors recognized as negative factors in this holistic approach to considering horse welfare and wellbeing [72]. Therefore, it is important to consider various anthropomorphic effects, such as the potential to influence an individual’s sense of empathy and perspectives about horse welfare and wellbeing when communicating with horse carers [30,68].

Other results in Theme One supported employing anthropomorphism to influence human behavior changes to improve horse welfare and wellbeing. Respondents identified the need to incorporate the topic of anthropomorphism into education programs. The finding resonates with reports of zoo communication programs utilizing anthropomorphism to engage the public on animal conservation and wellbeing messages [7,43]. The results are also commensurate with Thompson and Clarkson’s [30] recommendations for carefully using anthropomorphism in communication and education to engage horse owners. We suggest that horse sector education programs about assessing animal wellbeing remain incomplete unless the inclusion of the risks posed by the anthropomorphism of the horse’s mental experience is clearly elucidated to enable integration into day-to-day practice. Addressing the potential consequences of anthropomorphism requires urgent attention because adopting the Five Domains Model is becoming widespread throughout the horse sector, led by organizations including the Fédération Equestre Internationale and the International Federation of Horse Racing Authorities [73,74]. The tendency to anthropomorphize is prevalent in society; therefore, it is essential to build capacity among horse sector participants to actively manage its effects by adopting critical approaches and step-by-step methods for making inferences about equine mental experiences [3,13,18].

In Theme Two, the results indicated that situations may arise where experienced horse sector participants utilize elements of critical anthropomorphism. The results identified a perception that individuals had a duty to make decisions based on the horse’s species-specific requirements and instincts. Respondents recognized anthropomorphism as a factor when people infer the mental experiences of horses and make decisions about horse care and management. They perceived that risks could be mitigated if the inferrer remained cognizant of the science relating to horses and reflected on practical knowledge about horse management, training, and performance practices gained through life experiences. This reflexive approach, indicated by respondents’ reflections on how human attitudes towards horses could impact the horse’s experiences and the need to question behavioral interpretations and how these might have consequences for horse welfare and wellbeing, indicated an active approach to managing anthropomorphism was occurring. These results resonate with Hogg [24], who argues that while occasionally personifying horses, elite riders acknowledged their horse’s history, biological features, and inferred mental experiences, resulting in an informed empathy towards their mount. Further research is needed to understand how anthropomorphism manifests when humans interact with horses, particularly in competitive scenarios.

Another finding in Theme Two was related to the effect of anthropomorphic bias resulting in the misinterpretation of a horse’s motivations or behaviors, with respondents using labels such as lazy or retaliatory. The results correspond with previous studies implicating anthropomorphism in labeling dog, cat [32,75], and horse [40,42,76] behaviors. This study’s results also showed that individuals might inappropriately perceive horses as moral agents and therefore hold horses responsible for choosing to, or not choosing to, behave in a certain way. The results resonate with authors who argue that some individuals attribute the human ethical principle of moral agency to animals and hold them responsible for their behaviors [1,33]. Further, in the current study, the results identified a recognition that individuals could use this reasoning to justify aversive attitudes toward horses and punishment. Attributing responsibility to an animal for behavior choices is considered inappropriate [77,78]. However, the current study indicates that assigning human-like reasoning to horses could be widespread within the sector. McVey [42] reports how riders attributed horses with traits such as defiance and adopted a forceful attitude before awarding punishment by the excessive use of the reins and spurs. Other anthropomorphic explanations of horses in this current study include portraying horses as enjoying competitions and justifying practices such as stabling, even though it was acknowledged that in these situations horses can display behaviors indicative of negative mental experiences. The findings align with other studies reporting anthropomorphized explanations, such as a dog’s love for agility events or a Thoroughbred’s love of racing [65,79,80]. The examples of misinterpreting horse motivations and behaviors provided by respondents in this study align with Domain Four of the Five Domains Model [13]. This alignment then provides an opportunity to apply the Model to assess the impact of these misinterpretations on horse welfare and wellbeing. The value of this approach is that the Model can be contextualized and applied to a range of horse activities to assist with the assessment and management of anthropomorphic attitudes and impacts.

The Five Domain Model for animal welfare assessment invites humans to consider and evaluate animal welfare and wellbeing from the animal’s perspective [13]. If uncritical and unmanaged approaches to anthropomorphism prevail, it can negatively influence human attitudes toward horses and the interpretation of their behaviors and the subsequent making of inferences, rendering the application of the Model ineffective. Further, it can lead to faulty decision making regarding management, training, and performance practices, increasing the risk of negative welfare and wellbeing outcomes for horses [13]. Therefore, active approaches to managing anthropomorphism critically are required to ensure that the inferences regarding the horse’s subjective affective experiences are made with a higher level of confidence [11].

### 4.1. Organizations—Recognition, Managing, and Monitoring the Effects of Anthropomorphism

In this study, there was a perception that anthropomorphism was a common phenomenon, and the consequences for horses may not always be easy to identify. The authors are unaware of comparable investigations into the general public’s attitudes toward anthropomorphism and its application to horses or horse-related activities. When considering the effects of anthropomorphism as beneficial or detrimental to a horse’s wellbeing, individuals referred to their extensive experience and knowledge of horse management, training, and performance practices and to scientific evidence. The results of this study support the authors’ proposal that a cautious, critical approach to anthropomorphism has a place in animal welfare and wellbeing policy and practice.

#### Checklist to Assist Horse-Related Organizations in Managing Anthropomorphic Effects

We have constructed a checklist tool to assist horse-related organizations with the practical translation of the results of this study to manage anthropomorphic effects (Table 1). The checklist aligns statements about anthropomorphism with this study’s results and literature from animal science, ethics, and philosophy. The checklist is a tool to assist organizations in engaging with participants about anthropomorphism and raise awareness about its potentially harmful and beneficial aspects regarding horse welfare and wellbeing. The tool can inform communication strategies, promoting a more informed human connection to horses in a way that does not compromise their welfare. It can also be utilized to check for potential anthropomorphic effects when evaluating horse-related welfare and wellbeing in policies and practices. The first column proposes a checklist to recognize, monitor, and manage anthropomorphism consisting of three interrelated statements, which are informed by the results of this study and by the literature. The statements are (1) potential for anthropomorphic effects when inferring horse mental experiences; (2) potential for anthropomorphic effects on animal management, training, and performance practices; and (3) considering anthropomorphism when decision making and reviewing, implementing, and monitoring policies. The second column summarizes this study’s results, synthesizing horse sector participants’ viewpoints on anthropomorphism, horse welfare, and wellbeing. The third column provides examples of potential harmful effects identified in the results and provides examples of supporting evidence from the literature in science, ethics, and philosophy. The fourth column provides examples of situations where anthropomorphism, applied critically or used with caution, might have beneficial effects. Organizations can customize each column to meet their requirements. The alignment framework does not presuppose that anthropomorphizing is good or bad, as there are many factors to consider, such as the propensity of an individual to anthropomorphize, cultural influences, the human’s perception of their relationship with a horse, and the use of critical approaches [1,3,22].

### 4.2. Limitations

This study is qualitative and subjective [49,55]. Researcher positionality was managed through methods that included the employment of reflexive practices and the use of the Five Domains Model to support analysis and interpretation [13,56]. The ‘no’ responses to the close-ended question asking if anthropomorphism could influence horse welfare did not progress to the subsequent open-ended question asking how anthropomorphism related to horse welfare. This is a limiting factor because the opinions of horse sector participants who deny anthropomorphism or may have been unaware of the potential consequences, harmful or beneficial, for horse welfare and wellbeing were not collected. These opinions may have provided further insights into the extent of other issues relating to anthropomorphism, such as anthropomorphism denial [18]. Future research is required to understand the influence of anthropomorphism on horse welfare and wellbeing in the context of horse-related organizations and activities, specifically when humans interact with horses.

## 5. Conclusions

This study investigated the attitudes of experienced horse sector participants toward the concept and application of anthropomorphism and its actual and potential effects or perceived impacts on horse welfare and wellbeing. The results suggested that participants understood the complexities of anthropomorphism and its potential consequences, both beneficial and detrimental, for horse wellbeing. The findings reveal that anthropomorphic effects ranged from the attribution of human moral agency to horses, which could result in detrimental outcomes such as punishment, to creating a sense of increased empathy and motivation in people, which could result in beneficial outcomes such as providing better care for horses. The results from this study highlighted the complexities surrounding anthropomorphism and how it can impact the inferring of equine mental experiences and making decisions about a horse’s welfare and wellbeing state. The authors propose that a cautious, critical approach to anthropomorphism can have a role in aspects of horse welfare and wellbeing and could mitigate anthropomorphism-related risks when interpreting horse behaviors. The study results are important because adopting the Five Domains Model for animal welfare assessment requires subjective evaluation of an animal’s welfare and wellbeing state where risk associated with anthropomorphizing is a factor. An alignment framework is proposed to assist organizations in managing risks associated with the potential for anthropomorphic effects to influence policy and practices. Organizations can utilize the framework as a checklist when reviewing policies and practices to assist with managing and monitoring the potential effects of anthropomorphism. The proactive management of anthropomorphism can mitigate risks associated with the misinterpretation of horse behaviors, a potential risk for the sector’s social license to operate.

## Figures and Tables

**Table 1:** A checklist for horse-related organizations to translate the results of this study and guide the consideration of anthropomorphism in policy and practice. Column 1: the checklist proposes three interrelated statements developed from this study’s results. Column 2: horse sector participant viewpoints from this study. Column 3: potential harmful effects based on the results of this study supported by example references from the literature. Column 4: potential beneficial effects based on the results of this study supported by example references from the literature.

Checklist Items (Developed from the Results)	Horse Sector Participant ViewPoints from This Study	Manage Potential Harmful Effects (Results and Literature Examples)	Manage Potential Beneficial Effects (Results and Literature Examples)
1. Check for anthropomorphic effects when interpreting behaviors and inferring horse mental experiences.	Horse mental experiences can be inferred from observing and interpreting horse behaviors. Anthropomorphic effects can influence the interpretation of horse behaviors.	Potential harms include misinterpreting behaviors and misinformed inferences about equine mental experiences [6,13,34,36,70,75,81].	Applied critically, anthropomorphism can serve as a pre-step when making inferences about horse mental experiences [2,11,15,18,20,22,23,24].
2. Check for anthropomorphic effects when selecting horse management, training, and performance practices and when evaluating outcomes for horse welfare and wellbeing.	Potential beneficial effects include a sense of empathy and motivation to provide better horse care, which organizations can utilize in communication. Potential harms include misinterpreting species-specific needs and behaviors.	Misinformed behavioral evaluations may lead to a poor selection of practices, such as over-blanketing and punishment of horses [9,32,39,41,42].	Potential beneficial effects include an increased sense of empathy and motivation to build connections and a moral duty to provide better horse care [8,31,40].
3. Check for anthropomorphic effects when decision making and implementing and monitoring horse welfare and wellbeing policies.	Potential harms and benefits in group decision making and policies. It may influence public opinion.	Potential to influence or change public opinion [10,58,82,83,84]. Potential effects on policy [26,84].	Anthropomorphic effects can be utilized when communicating to motivate behavior changes for horse welfare and wellbeing [7,30,43,44,45,46].

## Data Availability

The data supporting this study’s findings are not publicly available due to privacy or ethical restrictions.
